# Reliability and reproducibility of the modified thoracolumbar injury classification and severity score and the thoracolumbar AO spine injury score for guiding surgical decision-making in thoracolumbar fractures

**DOI:** 10.3389/fsurg.2026.1828906

**Published:** 2026-06-26

**Authors:** Bing Wu, Jiale Zhang, Han Zhang, Junwei Feng, Jiayi Dou, Binbin Tang, Chen Chen, Liqiang Dong, Lianguo Wu, Weina Wang, Zhongcheng An, Tingyuan Lai

**Affiliations:** 1 The Second School of Clinical Medicine, Zhejiang Chinese Medical University, Hangzhou, Zhejiang, China; 2 Department of Orthopedics, The Second Affiliated Hospital of Zhejiang Chinese Medical University, Hangzhou, Zhejiang, China; 3 Department of Anesthesiology, The Second Affiliated Hospital of Zhejiang Chinese Medical University, Hangzhou, Zhejiang, China; 4 Department of Spine Surgery, Xinhua Hospital, Shanghai Jiaotong University School of Medicine, Shanghai, China

**Keywords:** mTLICS, reliability, reproducibility, thoracolumbar fracture, TL AOSIS

## Abstract

**Objective:**

To evaluate the reliability and reproducibility of the modified Thoracolumbar Injury Classification and Severity Score (mTLICS) and the Thoracolumbar AO Spine Injury Score (TL AOSIS) in guiding surgical decision-making for thoracolumbar fractures, and to investigate factors associated with observer agreement.

**Methods:**

A retrospective analysis was conducted on 100 thoracolumbar injury patients treated at the Second Affiliated Hospital of Zhejiang Chinese Medical University from January 2021 to December 2023, all with complete preoperative imaging data. The cohort included 64 males and 36 females, aged 25–55 years (mean 41.3 ± 6.9 years). Six evaluators independently assessed the anonymized cases on two occasions separated by a 4-week interval. Weighted Cohen's kappa coefficients were used to evaluate interobserver reliability and intraobserver reproducibility.

**Results:**

For TL AOSIS, interobserver/intraobserver kappa values 0.706/0.687 for fracture morphology, 0.906/0.942 for neurological status, 0.869/0.879 for tension band injury, and 0.736/0.732 for surgical recommendation, respectively. For mTLICS, interobserver/intraobserver kappa values were 0.773/0.763 for fracture morphology, 0.878/0.894 for neurological status, 0.716/0.721 for tension band injury, 0.837/0.845 for disc injury, and 0.702/0.685 for surgical recommendation, respectively. Significant differences (*P* < 0.05) were observed between systems in fracture morphology and tension band injury assessments, while no significant differences (*P* > 0.05) were found in neurological status evaluations.

**Conclusion:**

Both TL AOSIS and mTLICS demonstrate good reliability and reproducibility in guiding surgical decision-making for thoracolumbar fractures.

## 1 Introduction

Thoracolumbar fractures are among the common traumatic injuries in orthopedics. As the thoracolumbar region serves as a pivotal segment of the human spine and bears the majority of body weight, thoracolumbar fractures often result in significant functional impairment in patients (1). Without proper diagnostic and therapeutic guidance, severe sequelae such as kyphotic deformity, intervertebral disc degeneration, and chronic low back pain may develop (2, 3). Consequently, the selection of treatment strategies for thoracolumbar fractures remains a topic of significant clinical interest. Current research has focused largely on classification and scoring systems such as the Thoracolumbar AO Spine Injury Score (TL AOSIS) (4) and the Thoracolumbar Injury Classification and Severity Score (TLICS) (5). Some researchers have noted that compared with TL AOSIS, TLICS tends to favor conservative management in selected thoracolumbar fracture cases (6). However, in some patients, conservative management may lead to unsatisfactory clinical outcomes and a subsequent need for delayed surgical intervention (7). With advancements in biomechanical research, the role of intervertebral discs in spinal stability has garnered increased attention (8, 9). Building upon this, Lu et al. (10) proposed a modified Thoracolumbar Injury Classification and Severity Score (mTLICS) by reducing the scoring weight assigned to tension band injuries in the original TLICS and incorporating an intervertebral disc injury classification. Nevertheless, as mTLICS is a relatively recent development, its clinical validity, reliability, and reproducibility require further verification through extensive clinical data. To address this gap, this study retrospectively analyzed data from 100 patients with thoracolumbar injuries treated at the Department of Orthopedics, the Second Affiliated Hospital of Zhejiang Chinese Medical University between January 2021 and December 2023, all of whom had complete preoperative imaging records. The reliability and reproducibility of TL AOSIS and mTLICS in guiding surgical decision-making for thoracolumbar fractures were evaluated, and factors associated with agreement in these scoring systems were explored, aiming to provide evidence-based insights for clinical practice.

## 2 Materials and methods

### 2.1 Study population

A total of 100 patients with thoracolumbar fractures and complete preoperative imaging data admitted to the Department of Orthopedics at our institution between January 2021 and December 2023 were included in this study. The cohort comprised 64 males and 36 females, aged 25–55 years (mean ± SD: 41.3 ± 6.9). All cases involved single vertebral fractures. Imaging data included preoperative anteroposterior and lateral thoracolumbar radiographs, CT scans with three-dimensional reconstructions, and MRI. All imaging materials were anonymized and stripped of any classification-related annotations. This study was approved by the Ethics Committee of Zhejiang Chinese Medical University Second Affiliated Hospital.

Morphological assessment of thoracolumbar fractures was performed using anteroposterior and lateral radiographs, CT with three-dimensional reconstructions, and MRI. Posterior tension band/posterior ligamentous complex (PLC) integrity was assessed primarily on sagittal fat-suppressed T2-weighted or STIR images, with axial T2-weighted images serving as supplementary sequences, in accordance with previous MRI-based studies of PLC injury (11, 12). During image interpretation, particular attention was paid to the continuity and signal characteristics of the supraspinous ligament, interspinous ligament, ligamentum flavum, and facet capsule complex. For the purpose of between-system comparison, posterior tension band injury status was classified into three categories—intact, indeterminate, and definite injury—according to prior literature and the AO Spine concept of indeterminate tension band injury (11). Intact status was defined as the absence of abnormal signal change, widening, or structural discontinuity in the posterior ligamentous structures. Indeterminate injury was defined as subtle or equivocal hyperintense signal change, localized edema, or mild widening without definite fiber discontinuity. Definite injury was defined as clear structural discontinuity, frank rupture, marked widening, or pronounced edema-like hyperintense signal indicating posterior tension band failure (13). When MRI findings were equivocal and did not demonstrate definite structural disruption, the lesion was classified as indeterminate rather than definite injury according to the predefined study criteria.

MRI criteria for intervertebral disc injury were assessed in the cranial and caudal discs adjacent to the fractured vertebra using sagittal T1-weighted, T2-weighted, and fat-suppressed T2-weighted/STIR sequences. According to the MRI-based classification described by Sander et al. (14), disc lesions were first categorized into four grades (0–3) based on signal and morphologic alterations. Grade 0 indicated no signal or morphologic abnormality. Grade 1 represented disc edema with preserved morphology, characterized mainly by hyperintense signal on T2-weighted/STIR images. Grade 2 denoted disc rupture with intradiskal structural injury and signal alteration suggestive of intradiskal bleeding. Grade 3 indicated disc infraction into the vertebral body, annular tear, or herniation/endplate involvement (14). For mTLICS scoring, these MRI grades were subsequently collapsed into three categories: no injury (grade 0, 0 points), mild injury (grade 1, 1 point), and moderate-to-severe injury (grades 2–3, 2 points), consistent with the mTLICS framework (10).

#### 2.1.1 Inclusion criteria

(1) Traumatic single-level thoracolumbar fracture patients confirmed by initial imaging assessment; (2) Availability of complete clinical and imaging datasets, including complete preoperative imaging data: Preoperative anteroposterior/lateral radiographs of the thoracolumbar spine, CT with three-dimensional vertebral reconstruction; (3) MRI scans.

#### 2.1.2 Exclusion criteria

(1) Patients with multisegmental or established thoracolumbar fractures (e.g., ≥2 vertebral levels involved); (2) Non-traumatic thoracolumbar fractures, including pathological fractures (spinal tumor-associated fractures, infection-related fractures, or osteoporotic fractures); (3) Patients with pre-existing neurological impairments prior to the fracture event; (4) Patients with concomitant fractures of the lower extremities, pelvis, or other body regions; (5) Patients with incomplete clinical or imaging datasets (e.g., missing preoperative MRI or CT scans).

### 2.2 Imaging evaluation

Six spine surgeons (three junior and three senior) underwent standardized training on TL AOSIS and mTLICS scoring in the conference room of the Second Department of Orthopedics. After training and calibration, each surgeon independently reviewed the anonymized imaging datasets from 100 thoracolumbar fracture cases. Four weeks later, the same evaluations were repeated with randomized case sequences. A non-participating physician collated the data and analyzed the reliability and reproducibility of both scoring systems in guiding surgical decisions. For any patient, a discrepancy was recorded if any disagreement existed among the six surgeons regarding classification, scoring, or surgical recommendation during either evaluation round. Discrepancy factors were statistically analyzed to identify factors associated with disagreement.

TL AOSIS (Table 1) comprises three domains: fracture morphology, neurological status, and patient-specific modifiers, with a maximum score of 13 points. A total score < 4 points recommends non-surgical management; 4–5 points allow treatment selection based on patient preference or surgeon expertise; and >5 points indicate surgical intervention (15). The mTLICS (Table 2) evaluates four domains: fracture morphology, neurological injury, tension band integrity, and intervertebral disc injury. A total score <4 points recommends non-surgical management; 4 points permit non-surgical or surgical options depending on patient/surgeon preference; and >5 points indicate surgical treatment (10).

**Table 1:** AO thoracolumbar injury classification system.

Fracture classification	Subclassification	Score
A-Type	Compression fractures	
A0	Spinous/transverse process fracture	0
A1	Wedge fracture	1
A2	Split fracture	2
A3	Incomplete burst fracture	3
A4	Complete burst fracture	5
B-Type	Tension band injury	
B1	Posterior column injury (bony dominant)	5
B2	Posterior column injury (ligament dominant)	6
B3	Disc-mediated anterior column injury	7
C-Type	Translation/rotational displacement	8
Neurological status		
N0	Normal neurological function	0
N1	Transient neurological dysfunction	2
N2	Radiculopathy (symptoms/signs)	2
N3	Incomplete spinal cord/cauda equina injury	3
N4	Complete spinal cord injury	3
NX	Unable to assess due to head trauma, sedation, or multi-system injuries	
Patient-Specific Modifiers		
M1	Indeterminate tension band injury confirmed by MRI/clinical exam	1

**Table 2:** Modified thoracolumbar injury classification and severity score system.

Injury Type	Features	Score
Morphological Injury	Compression	1
	Burst fracture	2
	Displacement/rotation	3
	Distraction	4
Neurological Impairment	No neurological deficit	0
	Nerve root injury	2
	Complete spinal cord/conus medullaris injury	2
	Incomplete spinal cord/conus medullaris injury	3
	Cauda equina injury	3
Posterior Ligamentous Complex (PLC) Injury	Intact	0
	Suspected injury	1
	Injury	2
Intervertebral Disc Injury Status	No injury	0
	Mild injury	1
	Moderate-to-severe injury	2

### 2.3 Evaluation process

All patients were classified and scored using both the TL AOSIS and mTLICS systems. Data from two independent evaluations by six surgeons across both scoring systems were collected and summarized. The weighted Cohen's kappa coefficient was employed to assess interobserver reliability for the following parameters: fracture morphology classification, neurological status, tension band injury grading, intervertebral disc injury classification, treatment recommendations derived from both systems, and reliability among observers. Intraobserver reproducibility was determined by comparing the classification results from the same observer across the two evaluation rounds. Discrepant cases were statistically analyzed to identify factors associated with disagreement in classification, scoring, and surgical decision-making.

### 2.4 Statistical analysis

Statistical analyses were performed using SPSS 25.0 software. Weighted Cohen's kappa statistics were calculated, and mean kappa values were used to summarize the strength of agreement between observers and within the same observer across the two assessments. The kappa coefficient ranges from −1 to +1, where values >0 indicate meaningful agreement, with higher values reflecting stronger agreement. A kappa coefficient > 0.75 was interpreted as representing excellent agreement. The kappa coefficients for interobserver reliability and intraobserver reproducibility of fracture morphology classification, neurological status, tension band injury grading, and intervertebral disc injury classification derived from both scoring systems were compared among the six surgeons using independent t-tests. *P* < 0.05 was considered statistically significant.

## 3 Results

Across two evaluation rounds, six surgeons performed a total of 1,200 assessments (100 cases × 6 surgeons × 2 evaluations) for thoracolumbar fracture classification. Using the TL AOSIS system, Type A3 fractures were the most common, while Type B1 fractures were the least frequent. Morphological discrepancies were observed in 352 assessments (overall discrepancy rate: 29.3%). Discrepancies across the three major fracture types were distributed as follows: Type A (182 assessments, 51.7%), Type B (132 assessments, 37.5%), and Type C (38 assessments, 10.8%). Subtype analysis revealed discrepancies in Type A1 (18 assessments, 8.2%), A2 (22, 10.0%), A3 (30, 13.6%), and A4 (38, 17.3%); and Type B1 (8, 3.6%), B2 (60, 27.3%), and B3 (22, 10.0%). For treatment recommendations guided by TL AOSIS, 34 cases were advised for conservative management, 20 for optional surgical or conservative approaches, and 46 for surgical intervention. Using mTLICS, 32 cases were recommended for conservative management, 15 for optional approaches, and 53 for surgery.

### 3.1 Interobserver reliability analysis

For TL AOSIS, interobserver agreement yielded the following kappa values: 0.706 for fracture morphology classification, 0.906 for neurological status grading, 0.869 for tension band injury classification, and 0.736 for surgical recommendation.

For mTLICS, interobserver agreement yielded the following kappa values: 0.773 for fracture morphology, 0.878 for neurological status, 0.716 for tension band injury, 0.837 for intervertebral disc injury, and 0.702 for surgical recommendation.

### 3.2 Intraobserver reproducibility analysis

For TL AOSIS, intraobserver reproducibility yielded the following kappa values: 0.687 for fracture morphology classification, 0.942 for neurological status grading, and 0.876 for tension band injury classification. For mTLICS, intraobserver reproducibility yielded the following kappa values: 0.763 for fracture morphology classification, 0.894 for neurological status grading, 0.721 for tension band injury classification and 0.845 for intervertebral disc injury.

In fracture morphology classification, mTLICS demonstrated superior reliability and reproducibility compared to TL AOSIS (*P* < 0.05). Conversely, TL AOSIS exhibited significantly higher reliability and reproducibility in tension band injury classification than mTLICS (*P* < 0.05). For neurological status classification, both systems showed high reliability and reproducibility, with no statistically significant difference observed (*P* > 0.05). Table 3 presents the kappa comparisons of reliability and reproducibility for fracture morphology, neurological status, tension band injury classification, and intervertebral disc injury classification by six surgeons. Tables 4, 5 summarize the interobserver reliability and intraobserver reproducibility of TL AOSIS and mTLICS scoring systems, respectively.

**Table 3:** Kappa comparisons of reliability and reproducibility for fracture morphology, neurological Status, tension band injury classification, and intervertebral disc injury classification by Six surgeons.

Surgeons	Fracture morphology classification				Neurological status classification				Tension Band injury classification				Intervertebral disc injury classification	
	Interobserver reliability		Intraobserver reproducibility		Interobserver reliability		Intraobserver reproducibility		Interobserver reliability		Intraobserver reproducibility		Interobserver reliability	Intraobserver reproducibility
	TL AOSIS	mTLICS	TL AOSIS	mTLICS	TL AOSIS	mTLICS	TL AOSIS	mTLICS	TL AOSIS	mTLICS	TL AOSIS	mTLICS	mTLICS	mTLICS
1	0.615	0.725	0.627	0.712	0.895	0.822	0.892	0.856	0.895	0.698	0.886	0.692	0.794	0.814
2	0.622	0.717	0.638	0.723	0.872	0.838	0.906	0.853	0.802	0.761	0.835	0.674	0.806	0.798
3	0.671	0.752	0.661	0.726	0.937	0.842	0.921	0.852	0.832	0.743	0.827	0.713	0.815	0.825
4	0.754	0.826	0.712	0.809	0.925	0.925	0.965	0.914	0.954	0.759	0.915	0.802	0.885	0.869
5	0.795	0.802	0.761	0.816	0.870	0.946	0.983	0.898	0.938	0.791	0.943	0.839	0.863	0.902
6	0.779	0.816	0.723	0.791	0.937	0.895	0.985	0.871	0.925	0.692	0.868	0.798	0.859	0.862
t	−4.22		−3.87		1.43		2.01		6.12		4.85		-	-
P	0.0084		0.0116		0.2136		0.1012		0.0016		0.0045		-	-

**Table 4:** Interobserver reliability and intraobserver reproducibility of TL AOSIS scoring by Six surgeons.

Surgeons	Interobserver reliability		Intraobserver reproducibility	
	Percentage of agreement for classification scoring systems (%)	Kappa values	Percentage of agreement for classification scoring systems (%)	Kappa values
1	78.00 (73.00–82.00)	0.711 (0.489–0.643)	74.00	0.596
2	75.00 (71.00–79.00)	0.681 (0.536–0.715)	77.00	0.607
3	79.50 (74.00–85.00)	0.725 (0.583–0.767)	78.00	0.587
4	83.00 (80.00–86.00)	0.781 (0.708–0.795)	84.00	0.744
5	81.00 (77.00–85.00)	0.740 (0.713–0.767)	81.00	0.734
6	84.50 (82.00–87.00)	0.776 (0.745–0.816)	79.00	0.695

Surgeons 1–3 are junior surgeons; Surgeons 4–6 are senior surgeons. Kappa values > 0.75 indicate excellent agreement.

**Table 5:** Interobserver reliability and intraobserver reproducibility of mTLICS scoring by Six surgeons.

Surgeons	Interobserver reliability		Intraobserver reproducibility	
	Percentage of agreement for classification scoring systems (%)	Kappa values	Percentage of agreement for classification scoring systems (%)	Kappa values
1	75.50 (72.00–79.00)	0.716 (0.642–0.725)	73.00	0.646
2	77.50 (74.00–81.00)	0.696 (0.654–0.738)	76.00	0.681
3	72.00 (70.00–74.00)	0.661 (0.539–0.683)	72.00	0.576
4	84.50 (80.00–89.00)	0.825 (0.678–0.872)	87.00	0.746
5	79.00 (76.00–82.00)	0.717 (0.691–0.743)	79.00	0.704
6	82.00 (81.00–83.00)	0.765 (0.680–0.749)	81.00	0.715

Surgeons 1–3 are junior surgeons; Surgeons 4–6 are senior surgeons. Kappa values > 0.75 indicate excellent agreement.

### 3.3 Subgroup analysis based on evaluator experience

Subgroup analysis was conducted between senior and junior surgeons. For fracture morphology, the interobserver reliability of mTLICS was higher than TL AOSIS among both senior (0.814 vs. 0.776) and junior physicians (0.731 vs. 0.636). This difference in reliability between the two systems for fracture morphology was statistically significant (*P* < 0.05). Conversely, for tension band injury classification, TL AOSIS demonstrated superior reliability compared to mTLICS among both senior (0.925 vs. 0.823) and junior physicians (0.812 vs. 0.608), which was also statistically significant (*P* < 0.05).

## 4 Discussion

This study confirms that both mTLICS and TL AOSIS demonstrate good reliability and reproducibility in guiding surgical decision-making for thoracolumbar fractures, although each system shows distinct strengths. Our results showed that mTLICS had higher interobserver reliability and intraobserver reproducibility in fracture morphology classification, likely because of its simplified categorization, whereas TL AOSIS demonstrated superior reliability in the assessment of tension band injury. In addition, by incorporating intervertebral disc injury assessment, mTLICS tended to recommend surgical intervention more readily in certain borderline cases. Together, these findings suggest that the two systems provide different but complementary advantages in the evaluation of thoracolumbar fractures.

### 4.1 Fracture morphology classification

Overall, mTLICS demonstrated higher agreement than TL AOSIS in the assessment of fracture morphology. A likely explanation is that mTLICS adopts a simplified four-category framework, whereas TL AOSIS uses a more detailed nine-category morphological classification. This simpler structure may make mTLICS easier to apply consistently across evaluators, particularly in routine clinical settings. However, the greater simplicity of mTLICS may also reduce the granularity of morphological description. Therefore, although mTLICS may offer better practical consistency, TL AOSIS may still provide additional value when a more detailed characterization of complex fracture patterns is required.

In the TL AOSIS system, the number of inconsistent cases for type A fractures was 182 (51.7%). This does not indicate that type A fractures are more difficult to judge; rather, this situation primarily arises because type A fractures constituted the largest proportion of the sample in this study, resulting in the highest number of inconsistent cases. Among type B fractures, type B2 fractures had the highest number of inconsistent determination cases, totaling 60 (27.3%). This may be related to the difficulty of recognizing the imaging features of type B2 injuries, which involve posterior ligamentous structures. When proposing the TLICS system, Vaccaro et al. (5) also noted that the imaging features of type B injuries often generate controversy, as they involve the assessment of dynamic stability of posterior column structures, which may easily lead to divergences among different observers. Abedi A et al. (16) also pointed out that the interobserver agreement for type B fractures was significantly lower than that for type A or C fractures, likely due to the greater difficulty in assessing ligamentous structures in type B2 fractures.

From a clinical perspective, the higher reliability of mTLICS morphology classification may be particularly useful in emergency triage, multidisciplinary communication, and settings involving less-experienced surgeons, because a simpler framework is easier to apply consistently. However, this simplification may come at the cost of reduced granularity in describing complex injury patterns. Therefore, although mTLICS may improve practical usability, TL AOSIS may still offer additional value when a more detailed morphological characterization is needed for complex fractures.

### 4.2 Neurological status classification

For neurological status classification, both TL AOSIS and mTLICS demonstrated consistently high agreement, with no significant difference observed between the two systems. This finding suggests that neurological assessment is a relatively stable component of thoracolumbar fracture evaluation when standardized neurological documentation and clinical history are available. Because neurological status directly affects treatment urgency and prognosis, the high consistency observed in this domain supports the clinical utility of both systems in routine decision-making.

Thoracolumbar fractures with neurological injury are relatively common in clinical practice (17). Without proper diagnostic and treatment guidance, they may lead to permanent neurological damage or even paraplegia, significantly affecting patients' quality of life (18). Therefore, assessment of neurological injury is essential in the diagnostic process of thoracolumbar fractures.

### 4.3 Tension band injury classification

Accurate assessment of tension band injury is clinically important because posterior tension band failure indicates mechanical instability and may shift borderline injuries toward surgical stabilization (19). In the present study, TL AOSIS showed significantly higher agreement than mTLICS in this domain. The superior reliability and reproducibility of TL AOSIS may be attributed to its more detailed description of fracture morphology classification, which can partially reflect the presence or absence of tension band injuries and assist clinicians in interpretation. Radiologically, the close adjacency of posterior tension band structures to surrounding tissues may lead to overlap or artifacts on MRI (20).

Additionally, MRI-based assessment of posterior tension band injury remains inherently challenging. Post-traumatic signal alterations may overlap with normal physiological findings or other nonspecific abnormalities, making it difficult to distinguish edema, partial fiber disruption, and early-stage injury (12, 21). Because mTLICS categorizes posterior tension band injury as no injury, suspected injury, or definite injury, its application still relies partly on subjective interpretation (22).

As detailed in our results (Section 3.3), professional expertise significantly influenced the reliability of both scoring systems. For fracture morphology, mTLICS showed higher reliability than TL AOSIS among both senior and junior physicians, suggesting that its simplified four-category framework may be easier for less-experienced raters to apply consistently. By contrast, TL AOSIS showed higher agreement in the assessment of tension band injury, particularly among senior surgeons, indicating that experience remains critical for consistent MRI-based interpretation of posterior ligamentous injury. Clinically, this difference is most relevant in borderline thoracolumbar fractures, particularly in neurologically intact patients in whom fracture morphology alone does not clearly determine whether the injury is mechanically stable or unstable. In such cases, the assessment of posterior tension band may become a key factor in deciding whether conservative treatment is sufficient or surgical stabilization should be recommended. Therefore, higher reproducibility in tension band injury assessment may reduce interobserver variability in treatment recommendations and provide more stable decision support across surgeons. This interpretation is consistent with our subgroup findings showing that TL AOSIS achieved higher agreement than mTLICS in tension band assessment among both senior and junior surgeons. However, this advantage should not be interpreted as evidence of superior diagnostic accuracy, because MRI-based evaluation of posterior ligamentous injury remains challenging and partly subjective.

However, this apparent advantage of TL AOSIS should be interpreted cautiously. Previous literature has shown that posterior ligamentous complex assessment remains controversial and imperfect even among experienced observers. Van Middendorp et al. reported substantial variability in the precision and accuracy of PLC detection and emphasized that the prognostic value of PLC injury remains insufficiently established (21). Likewise, Canseco et al. found that even among 22 spine trauma experts, agreement for borderline AO patterns such as A3/A4 injuries and for the M1 modifier was limited (23). In this context, the lower agreement of mTLICS in tension band injury classification should not be interpreted simply as inferior performance. Rather, it may reflect the conceptual design of mTLICS, which reduces the weighting of posterior tension band injury and balances it against other MRI-derived indicators of instability, thereby potentially reducing overreliance on a single difficult-to-interpret variable in equivocal cases (22).

### 4.4 Intervertebral disc injury classification

In this study, the intervertebral disc injury component of mTLICS showed good agreement, supporting the feasibility of incorporating disc status into the evaluation of thoracolumbar fractures. Clinically, MRI is sensitive to disc injury and provides imaging features that are relatively amenable to interpretation, which may help explain the satisfactory consistency observed in this domain. These findings also support the rationale for including disc injury in scoring systems intended to identify mechanically vulnerable fractures that may otherwise appear borderline by morphology alone.

The intervertebral disc, composed of the annulus fibrosus, nucleus pulposus, and cartilaginous endplates, functions to withstand pressure, distribute stress, and provide spinal flexibility (24). Multiple studies have shown that direct trauma to the human body often results in concurrent thoracolumbar fractures and intervertebral disc injuries (25, 26). Under normal conditions, intervertebral discs maintain spinal stability and mobility under varying loads. However, thoracolumbar fractures alter the biomechanical properties of the discs, thereby affecting spinal stability (27). In cases of mild disc injury, where disc material does not protrude into the fractured vertebra, conventional surgical procedures can effectively restore vertebral height and correct kyphotic deformity. Conversely, severe disc damage with protrusion of disc material into the fractured vertebra significantly increases the risk of delayed kyphosis (28). Therefore, incorporating disc injury assessment into scoring systems is a rational approach.

Cross-system analysis showed that mTLICS generated surgical recommendations more frequently than TL AOSIS (53 vs. 46 cases). This discrepancy is primarily driven by the incorporation of intervertebral disc injury scores in mTLICS. In certain borderline cases (e.g., neurologically intact burst fractures without definite tension band rupture), the presence of severe disc injury tipped the mTLICS score past the surgical threshold (>5 points). Given that unaddressed severe disc injuries can lead to delayed kyphosis, disc degeneration, and chronic discogenic low back pain (29, 30), mTLICS may provide a broader basis for surgical consideration in selected trauma patterns. This potential advantage should be balanced against the possibility that MRI-detected disc abnormalities may increase the number of patients classified above the operative threshold, even when the actual long-term benefit of surgery has not been fully established.

Overall, the clinical relevance of the observed between-system differences lies not in the isolated performance of each individual scoring domain, but in how each system characterizes borderline injuries in actual surgical decision-making. In neurologically intact patients with thoracolumbar fractures in whom fracture morphology alone does not clearly distinguish mechanical stability from instability, the higher reproducibility of TL AOSIS in assessing posterior tension band injury may help reduce interobserver variability and provide more stable support for decisions regarding operative vs. non-operative treatment. By contrast, in neurologically intact burst fractures without definite posterior tension band rupture but with substantial MRI-detected adjacent disc injury, mTLICS may be more likely to identify these cases as mechanically vulnerable patterns because disc injury contributes directly to the total score. This may partly explain why mTLICS showed a greater tendency to recommend surgery in our cohort. This interpretation is also consistent with the conceptual background of the two systems: TL AOSIS was developed as a severity metric to support treatment threshold determination within the AOSpine framework (15), whereas the revised AO thoracolumbar classification emphasizes morphology-based patterns of mechanical failure (31). In contrast, MRI-guided refinements such as mTLICS were introduced to capture instability-related findings that may not be fully reflected by morphology and neurological status alone, particularly in cases in which conventional systems provide equivocal guidance (22). Therefore, the practical difference between the two systems is not simply which one performs better overall, but which type of potentially unstable patient each system is more likely to identify at the borderline between conservative and surgical management. However, because this study was designed as a reliability and reproducibility study rather than an outcome study, the present findings should be interpreted as indicating which system shows higher agreement and reproducibility in specific domains, rather than proving that one system is definitively more accurate or associated with better long-term prognosis.

### 4.5 Limitations

This study has several limitations. First, it was a retrospective single-center study, which may have introduced selection bias and information bias. Because the assessments were based on archived clinical and imaging data rather than real-time clinical decision-making, the observed agreement may not fully reflect daily practice. Second, the sample size was relatively small, which may limit the statistical stability of the findings, particularly in subgroup analyses and less common fracture patterns. Third, only patients with single-level thoracolumbar fractures were included, while multilevel injuries and other complex fracture patterns were excluded; therefore, the generalizability of our findings is limited. Therefore, our findings should be interpreted with caution. Future large-scale, prospective, multicenter studies including more heterogeneous fracture patterns are needed to further validate the reliability and clinical applicability of both scoring systems.

## 5 Conclusions

Both TL AOSIS and mTLICS demonstrate satisfactory reliability and reproducibility for guiding treatment decisions in thoracolumbar fractures. Evaluator experience significantly influences assessment outcomes: mTLICS may be easier for junior surgeons to apply in morphology assessment, whereas TL AOSIS shows greater stability in evaluating tension band injury. Notably, by incorporating intervertebral disc injury assessment, mTLICS exhibits a lower threshold for recommending surgical intervention in specific borderline cases. This comprehensive approach may provide additional information relevant to long-term spinal stability. The choice of scoring systems should be individualized according to clinician experience and the patient's radiological characteristics. However, given the relatively small sample size and retrospective design of this study, these findings should be interpreted with caution and require further validation in large prospective multicenter studies.

## Data Availability

The raw data supporting the conclusions of this article will be made available by the authors, without undue reservation.
